# Stability prediction of early orthopedic treatment in Class III malocclusion: morphologic discriminant analysis

**DOI:** 10.1186/s40510-021-00379-z

**Published:** 2021-09-20

**Authors:** V. Paoloni, F. C. De Razza, L. Franchi, P. Cozza

**Affiliations:** 1 Department of Systems Medicine, University of Rome Tor Vergata, Rome, Italy; 2 Department of Surgery and Translational Medicine, University of Florence, Florence, Italy

**Keywords:** Class III malocclusion, Early orthopedic treatment, Dental cast analysis, Discriminant analysis

## Abstract

**Background:**

To evaluate morphologic differences between class III malocclusion success and failure treatment subjects in order to identify which variables are more predictive for long-term stability in early orthopedic treatment. In this retrospective study, 31 patients were enrolled from the Department of Orthodontics (Rome Tor Vergata). Inclusion criteria were as follows: white ancestry, class III malocclusion, mixed dentition, cervical stage (CS) 1-2, no pseudo-class III. Pre-treatment radiographic and cast records were collected. Each patient underwent rapid maxillary expansion/facial mask/bite block (RME/FM/BB) orthopedic treatment until correction. At T1 (permanent dentition, CS4), records were recollected. According to treatment stability, relapse group (RG, 19) and success group (SG, 12) were identified. Sagittal and vertical cephalometric and digital cast measurements were performed. Student’s *t* tests were used for statistically significant differences inter and intra groups. For discriminant analysis, relapse or success status was added to each patient’s T0 data.

**Results:**

At T0, RG showed larger upper anterior transversal width (*p* = 0.0266), while at T1 the upper anterior length was shorter than SG (*p* = 0.0028). Between T1 and T0, both groups showed larger upper anterior and posterior transversal widths. SG had greater upper anterior (*p* = 0.0066) and posterior (*p* = 0.449) sagittal length. RG presented larger lower anterior (*p* = 0.0012) and posterior (*p* = 0.0002) transversal widths, while there were no differences in SG lower arch. Discriminant analysis provided two predictive variables with an accuracy of 80.6%: upper anterior length and upper posterior length.

**Conclusion:**

A shorter and wider maxilla could be a predisposing factor for relapse and failure of the early orthopedic treatment of class III malocclusion patients. The absence of mandibular changes could be predictable for treatment success.

## Background

Treatment stability in class III malocclusion is a topic of crucial importance, as many factors are involved in prognosis and in long-term response. The etiology is wide-ranging and complex [1]: A synergic relationship between genetics and environment in class III malocclusion development is generally assumed [2]. However, these factors may act in isolation or to cancel each other out [1]. For this reason, it is accepted from scientific community that is necessary to treat these patients as soon as possible on pre-pubertal stage [2, 3]. Moreover, growth represents the main opponent to long-term stability in class III subjects [4]. Many studies found out that in these patients the growth spurt lasts longer than class I and class II adolescents [4, 5]. These changes affect especially the mandibular bone: mandibular growth occurs in class III subjects after the adolescent growth spurt; thus, there are dental compensation movements for the worsening skeletal discrepancy that accompany differential growth of the jaws [6, 7]. According to Chen et al. [8], the major factor that determines long-term successful treatment is not the maxillary response to forward traction, but the amount and direction of mandibular growth during and after adolescence. In these cases, knowledge of stability predictive variables is essential to plan the best therapy.

In 1966, Tweed [9] described two different class III malocclusion prognostic patterns. He identified a favorable pattern, characterized by a normal mandibular size, narrow and short maxilla, normal gonial angle and reduced vertical skeletal pattern, and on the other hand an unfavorable pattern, typified by a large protruded mandibula, narrow maxilla, obtuse gonial angle, increase vertical skeletal pattern, and hypertonic lower lip. Since then, many authors investigated prognostic factors useful as predictive stability variables, but their works generally focused on cephalometric analysis. The predictive stability variables most frequently observed are as follows: gonial angle, Wits appraisal, ramus length, lower incisors inclination relative to mandibular plane, mandibular plane angle, SNB angle, and CondAx-SBL [10–24].

Only few studies analyzed the predictive variables of treatment success in class III malocclusion on dental arches. In 1997, Franchi et al. [21] evaluated three linear transversal measurements (maxillary and mandibular deciduous intermolar width, transverse discrepancy) in 45 dental casts of patients with class III malocclusion in deciduous dentition, and they found that mandibular transverse width was a relapse predictor. Twenty years later, in 2017, Wendl et al. [22] examined the differences between successful and unsuccessful treated class III malocclusion patients on dental casts and found out that a narrower maxillary intermolar width could be an additional risk to relapse.

To our knowledge, no studies assessed the presence of predictive stability variables in dental arches with three-dimensional analysis after early orthopedic therapy. The most popular early orthopedic treatment protocol for class III malocclusion is the combination of rapid maxillary expansion (RME) followed by maxillary protraction with facemask (FM) [25].

For this reason, the aim of this study was to evaluate dental arches morphology in class III malocclusion subjects and to identify their morphologic differences by comparing a success and a failure treatment group after early orthopedic therapy.

## Methods

This project was approved by the Ethical Committee of the University of Rome Tor Vergata (Protocol number: 201/19), and informed consent was obtained from the patients’ parents.

In this retrospective study, a sample of 31 patients (19 males, 12 females, 8.3 years ± 5 months), who were treated consecutively with RME/FM protocol and with bite block (BB) from 2005 to 2014, was enrolled from the Department of Orthodontics at the University of Rome Tor Vergata. Inclusion criteria were white ancestry, class III skeletal malocclusion (ANB < 0°, Wits < −2 mm) characterized by an anterior crossbite or edge-to-edge incisal relationship, early mixed dentition phase, skeletal maturation between cervical stage (CS) CS1-CS2 evaluated by cervical stage valuation (CSV) method as described by Baccetti et al. [26], no pseudo-class III due to forced bite, class III familiarity, no previous orthodontic treatment, and no congenital diseases.

At pre-treatment phase (T0), radiographic records were acquired (panoramic and lateral cephalograms) and upper and lower dental casts were collected.

Every patient underwent the same treatment protocol. A bonded rapid maxillary expander (RME) with vestibular hooks for facial mask (FM) was placed on first upper molars. The screw was activated one turn a day until transversal overcorrection achievement. At the end of active expansion, FM was given for maxillary protraction with extra-oral elastics, delivering 400–500 g of force per side, with about 30 degrees of downward inclination relative to the occlusal plane and patients were instructed to wear the mask 14-16 h per day (night-time included). Moreover, to control the vertical dimension an acrylic bite-block appliance (BB) with occlusal lift planes was used [27] and each patient was asked to wear the functional appliance 22 h per day.

At the end of this first phase of treatment, all patients reached the class III malocclusion orthopedic correction, and they underwent routine recalls to follow-up treatment stability.

These patients were seen every 6 months until they reached a skeletal cervical maturation CS4 with all their permanent teeth erupted except for the third molars (mean age 14.5 years ± 5 months). Before starting the second phase of treatment (T1), radiographs and dental casts were acquired to assess orthopedic treatment stability, checking molar, and canine dental class, as reported by Wendl et al. [22], sagittal and vertical skeletal relationship on lateral cephalograms and esthetic characteristics of patient’s profile [20].

According to the treatment stability of early orthopedic therapy, two groups were identified: relapse group (RG) and success group (SG). RG (19 patients, 12M, 7F 12.1 ± 3 years) presented at T1 skeletal class III malocclusion (ANB < 0°, Wits < −2 mm), class III molar and canine relationship, unfavorable esthetical characteristic and needed for a new phase of therapy to achieve the correct occlusal relationship; on the contrary, SG (12 patients, 7M, 5F, 12.7 ± 2 years) presented class I occlusion and good esthetical characteristics.

**Fig. 1:**
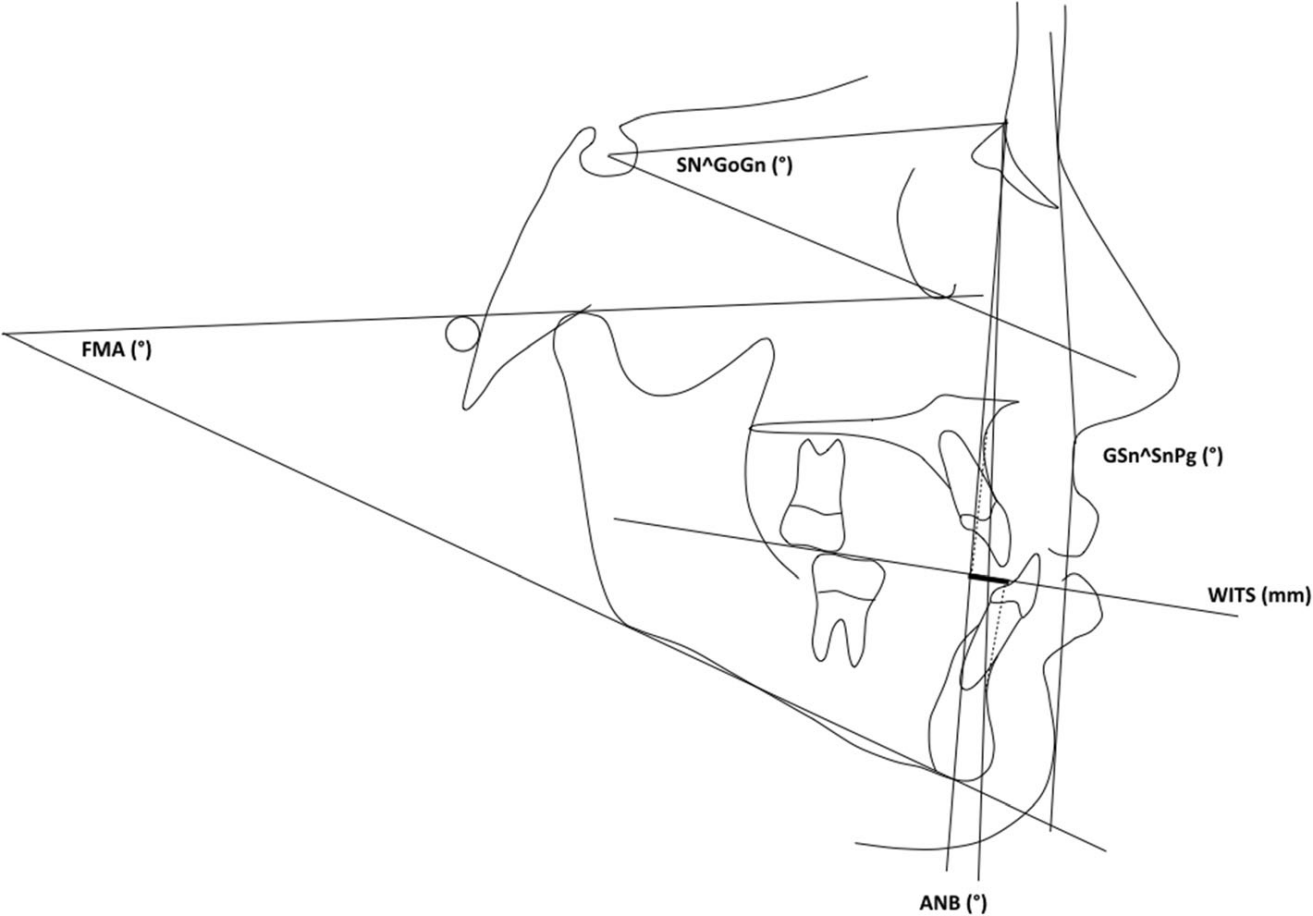
Angular and linear measurements performed at pre-treatment and post-treatment phase on lateral cephalometric radiographs

**Fig. 2:**
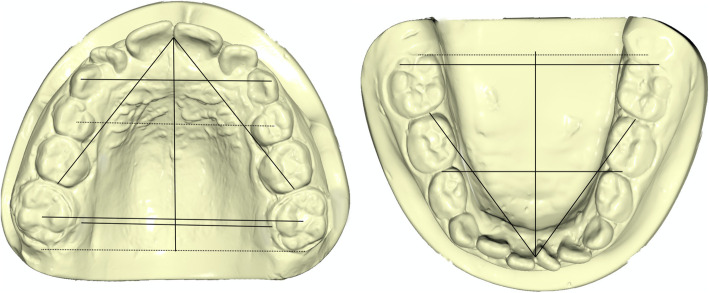
Transversal and sagittal linear measurements performed at pre-treatment and post-treatment phase on upper and lower digital casts

**Fig. 3:**
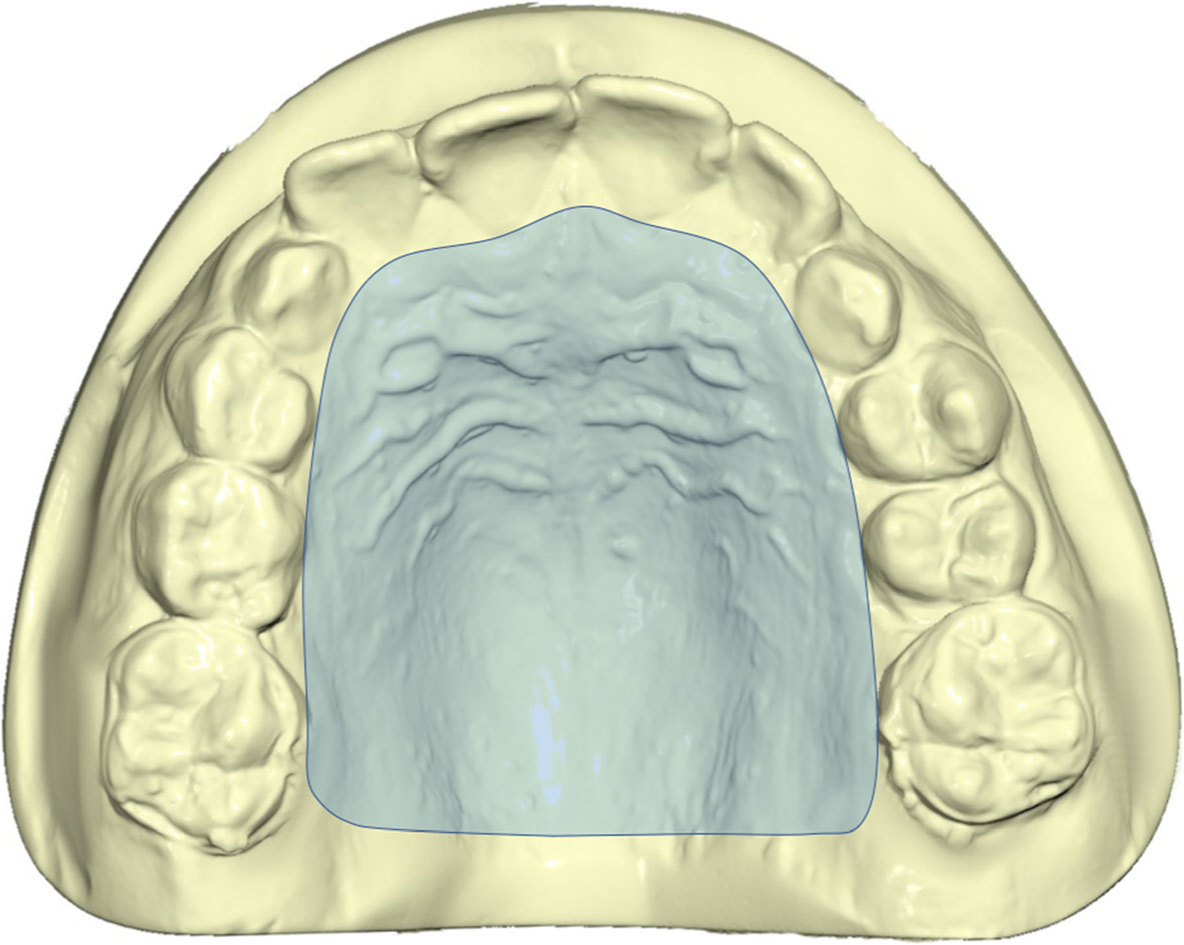
Graphic description of palatal area measurement, comprised within the crossing point between lingual groove and gingival border of both deciduous and permanent upper incisors, canines, premolars, and molars

On lateral cephalograms, linear measurements were performed to diagnose the class III skeletal malocclusion and evaluate patients’ divergence (ANB, Wits appraisal, FMA, and SN^GoGn) (Table 1) (Fig. 1). To evaluate the profile, the facial angle Glabella–Subnasale–Pogonion (G–Sn–Pg) was measured on the lateral radiographs and an acceptable profile was defined if it was equal to or smaller than 174 degrees; otherwise, the profile was considered unacceptable [20] (Table 1) (Fig. 1). Each dental cast was scanned through the extraoral scanner OrthoXscan (OrthoXscan; Dentaurum GmbH&co, Ispringen, Germany) and exported in a Standard Tessellation Language format (.stl). The digital casts were analyzed using a specific software (Viewbox, dHAL software, Kifissia, Greece): 32 points were identified and 11 linear measurements and the palatal area were evaluated [28, 29] (Table 1) (Figs. 2 and 3).

**Table 1:** Description of the measurements on lateral radiographs and dental casts

Measurements	Description
Upper anterior transversal width (UATW)	Distance between the cusp tips of upper deciduous or permanent canines.
Upper posterior transversal width (UPTW)	Distance between upper permanent molars intersection points between transversal and buccal grooves
Upper anterior sagittal length (UASL)	Line drawn from the palatal interincisal midpoint, perpendicular to the conjunction line between the centers of first premolars.
Upper posterior sagittal length (UPSL)	Line drawn from the palatal interincisal point, perpendicular to the conjunction line between the distal groove of first molars (or contact point between first and second molar).
Upper-molar alveolar width (UM-AW)	Distance between permanent upper first molar, considering the crossing point between lingual groove and gingival border.
Upper arch length (UAL)	Sum of right and left distances between the upper incisors most anterior point and second upper deciduous molar or permanent premolar and upper first permanent molar contact point.
Lower anterior transversal width (LATW)	In deciduous dentition, distance between the disto-labial cusp tips of lower first deciduous molars; in permanent dentition, distance between first and second premolars contact point.
Lower posterior transversal width (LPTW)	Distance between the disto-labial cusp tips of lower first permanent molars.
Lower anterior sagittal length (LASL)	Line drawn from the lingual interincisal midpoint, perpendicular to the conjunction line used to measure lower anterior transversal width.
Lower posterior sagittal length (LPSL)	Line drawn from the lingual interincisal point, perpendicular to the conjunction line used to measure lower intermolar width.
Lower arch length (LAL)	Sum of right and left distances between the lower incisors most anterior point and second lower deciduous molar or permanent premolar and lower first permanent molar contact point.
Palatal area (area)	Measure of palatal surface, comprised within the crossing point between lingual groove and gingival border of both deciduous and permanent upper incisors, canines, premolars and molars.
ANB	Angle between N-A and N-B
WITS appraisal (Wits)	Distance determined by the distance between the orthogonal projections of points A and B on the functional occlusal plane
FMA	Angle between Frankfurt plane and mandibular plane
SN^GoGn	Angle between Sella-Nasion and Gonion-Gnation
GSn^SnPg	Angle between Glabella-Subnasal and Subnasal-Pogonion

### Statistical analysis

In a pilot study, 10 patients were used to calculate the reproducibility and the sample size which indicated the need for approximately 22 patients to estimate the upper anterior transversal width with a 95% confidence interval (CI); a minimum difference of 2.5 mm and a standard deviation (SD) of 2.5 mm, with a power of 80%.

To determinate the method accuracy, measurements on digital dental casts were performed by one trained examiner with an experience of 5 years (FCDR) and repeated by the same approximately 15 days after. A paired *t* test was used to compare the two measurements (systematic error, *p* value < 0.05). In the presence of normally distributed data, descriptive statistics were calculated for each measurement in each group and significant between-group differences were tested with the independent sample Student’s *t* test. The level of significance was set at 5%.

Relapse or success status was added to preliminary patient treatment data. The measurements were examined using discriminant analysis, run under the IBM statistical package for the social sciences (IBM Corp. Released 2019. IBM SPSS Statistics for Windows, Version 26.0. Armonk, NY: IBM Corp). Discriminant analysis is a statistic model, specifically designed to widely separate two groups of subjects taken from the same population [30]. For this reason, this statistical process was used to identify a reliable variable in predicting success or relapse in early class III treatment. At first, all eligible variables were entered with a stepwise variable selection and then the number was progressively reduced by sequentially excluding the variables with minor contribution to the overall discrimination, to achieve a complete and correct separation between the two groups using the smallest number of variables as possible [30, 31].

## Results

No systematic error was found.

Descriptive statistics are reported in Table 2. The sample was made of 31 subjects (19M, 12F) in class III skeletal malocclusion (*ANB* = −0.878°; *Wits* = −6.306 mm) with a normodivergent pattern (*FMA* = 26.62°; *SN^GoGn* = 34.857°). According to treatment stability, two groups were identified, and intra-groups and inter-groups analysis were performed. At T0, no significant statistical differences were present between the two groups for vertical and sagittal cephalometric measurements. At T1, the cephalometric analysis showed a hyperdivergent pattern (*FMA* = 30.8°±3.8°; *SN^GoGn* = 38.9°±2.3°) with reduced values of ANB (1.3° ± 1°) and Wits (−11 mm ± 3.7 mm) in RG compared to SG (Tables 3 and 4).

**Table 2:** Descriptive statistics of the collected sample before treatment (T0)

*31 patients (19M, 12F, 8.3 years ± 5 months)*		
	Mean values	SD
FMA	26.62	3.5
ANB	−0.878	1.45
Wits	−6.306	4.21
Sn^GoGn	34.857	4.46

**Table 3:** Statistical comparisons between groups at T0: cephalometric analysis

	Relapse group (RG)—T1		Success group (SG)—T1		Diff.	*t* tests
	Mean	SD	Mean	SD		*P*
FMA	26.91	2.84	25.20	3.74	−1.71	0.1594
ANB	−0.86	1.29	−1.50	2.01	−0.64	0.2874
WITS	−8.64	4.08	−6.64	4.11	2.00	0.1953
SN^GoGn	35.73	3.80	33.8	4.42	−1.93	0.2060

**Table 4:** Statistical comparisons between groups at T1: cephalometric analysis

	Relapse group (RG)—T1		Success group (SG)—T1		Diff.	*t* tests	
	Mean	SD	Mean	SD		*P*	
FMA	30.8	3.8	25.5	4.32	−6.37	0.0073	**
ANB	1.33	1.03	2.8	2.3	1.35	0.158	
WITS	−11	3.71	−3.1	2.6	7.9	0.0025	**
SN^GoGn	38.9	2.3	33.9	3.5	−5.32	0.003	**

Focusing on dental measurements, at T0, the inter-groups statistical analysis presented difference between RG and SG in the upper anterior transversal width (UATW), which resulted larger in RG (32.02 mm ± 2.83 mm) than SG (29.2 mm ± 3.89 mm) (Table 5). At T1, a significant statistical difference was also found in the upper anterior sagittal length (UASL), which was shorter in RG (14.24 mm ± 3.22 mm) than in SG (18.24 mm ± 3.47 mm) (Table 6).

**Table 5:** Statistical comparisons between groups at T0: digital dental casts measurements

	Relapse group (RG)—T0		Success group (SG)—T0		Diff.	*t* tests	
	Mean	SD	Mean	SD		*P*	
UATW	32.02	2.83	29.2	3.89	−2.821	0.0266	*
UPTW	44.18	3.87	43.68	4.45	−0.5009	0.743	
UASL	13.95	0.97	14.08	2.73	0.1254	0.9769	
UPSL	34.63	4.5	36.79	3.73	2.163	0.1753	
UM-AW	33.21	3.37	32.95	3	−3.987	0.4767	
UAL	65.92	5.67	65.73	3.86	−0.1961	0.9171	
PArea	876.38	163.51	762.64	144.41	−113.7	0.0584	
LATW	27.12	3.62	26.41	2.49	−0.7164	0.553	
LPTW	48.06	3.19	47.56	1.9	−0.5048	0.6521	
LASL	15.53	4.88	18.47	3.48	2.940	0.0803	
LPSL	33.08	8.03	36.88	3.13	3.791	0.1311	
LAL	62.17	8.08	60.44	6.91	−1.732	0.4334

**Table 6:** Statistical comparisons between groups at T1: digital dental casts measurements

	Relapse group (RG)—T1		Success group (SG)—T1		Diff.	*t* test	
	Mean	SD	Mean	SD		*P*	
UATW	35.6	3.56	34.52	2.47	−0.7714	0.4922	
UPTW	48.35	2.63	46.59	1.75	−1.756	0.0509	
UASL	14.24	3.22	18.24	3.47	4	0.0028	**
UPSL	37.95	4.87	39.04	2.77	1.094	0.4856	
UM-AW	36.27	3.06	34.68	2.21	−1.593	0.129	
UAL	69.66	6.62	66.45	7.41	−3.213	0.2188	
PArea	969.05	274.09	821.58	126.95	−147.5	0.0923	
LATW	29.25	4.57	27.76	3.11	−1.489	0.3297	
LPTW	50.41	3.73	49.1	3.17	−1.311	0.3226	
LASL	16.41	5.32	19.06	2.81	2.653	0.1237	
LPSL	33.54	8.08	35.04	2.65	2.305	0.3493	
LAL	62.85	8.17	60.78	7.8	−2.069	0.4903	

Examining the intra-group differences T1-T0 (Table 7), both groups showed significant improvement in both anterior and posterior upper arch transversal dimensions due to the orthopedic maxillary expansion. In RG, both lower intercanine width (*p* = 0.0012) and lower intermolar width (*p* = 0.0002) increased at T1. On the contrary, SG showed a statistically significant improvement in the upper anterior sagittal length (*p* = 0.0066) and in the upper posterior sagittal length (*p* = 0.0449). The lower arch showed no statistically significant changes.

**Table 7:** Statistical comparisons of the T1-T0 changes in the RG and in the SG

	Relapse group (RG) T1-T0			Success group (SG) T1-T0		
	Diff T1-T0	*t* tests *P*		Diff T1-T0	*t* tests *P*	
UATW	3.58	0.0001	****	5.32	0.0006	***
UPTW	4.17	0.0001	****	2.91	0.0497	*
UASL	0.28	0.717		4.16	0.0066	**
UPSL	3.32	0.0569		2.25	0.0449	*
UM-AW	3.06	0.0004	***	1.73	0.1856	
UAL	3.74	0.0296	*	0.72	0.7055	
Area	92.67	0.1468		58.94	0.2261	
LATW	2.13	0.0012	***	1.35	0.1423	
LPTW	2.35	0.0002	***	1.54	0.1025	
LASL	0.88	0.3164		0.59	0.7076	
LPSL	0.46	0.6436		−1.84	0.3138	
LAL	0.68	0.731		0.34	0.8535	

Table 8 showed the discriminant analysis results. The discriminant analysis produced a model of two predictive variables, represented by:

- Upper posterior sagittal length (statistical significance = 0.027)
- Upper anterior sagittal length (statistical significance = 0.011)

**Table 8:** Discriminant analysis of predictive success variables in class III early orthopedic treatment

	Classification results							
Groups	Number of cases	Predicted group membership				Constants (Fisher’s linear discriminant function)	Predictive variables	Standardized canonical discriminant function coefficients
		Relapse group (RG)		Success group (SG)				
		No.	%	No.	%			
Relapse group (RG)	19	15	78.9	4	21.1	−33.361	*Upper posterior sagittal length*	1.117
Success group (SG)	12	2	16.7	10	83.3	−29.897	*Upper anterior sagittal length*	−1.266

**Fig. 4:**
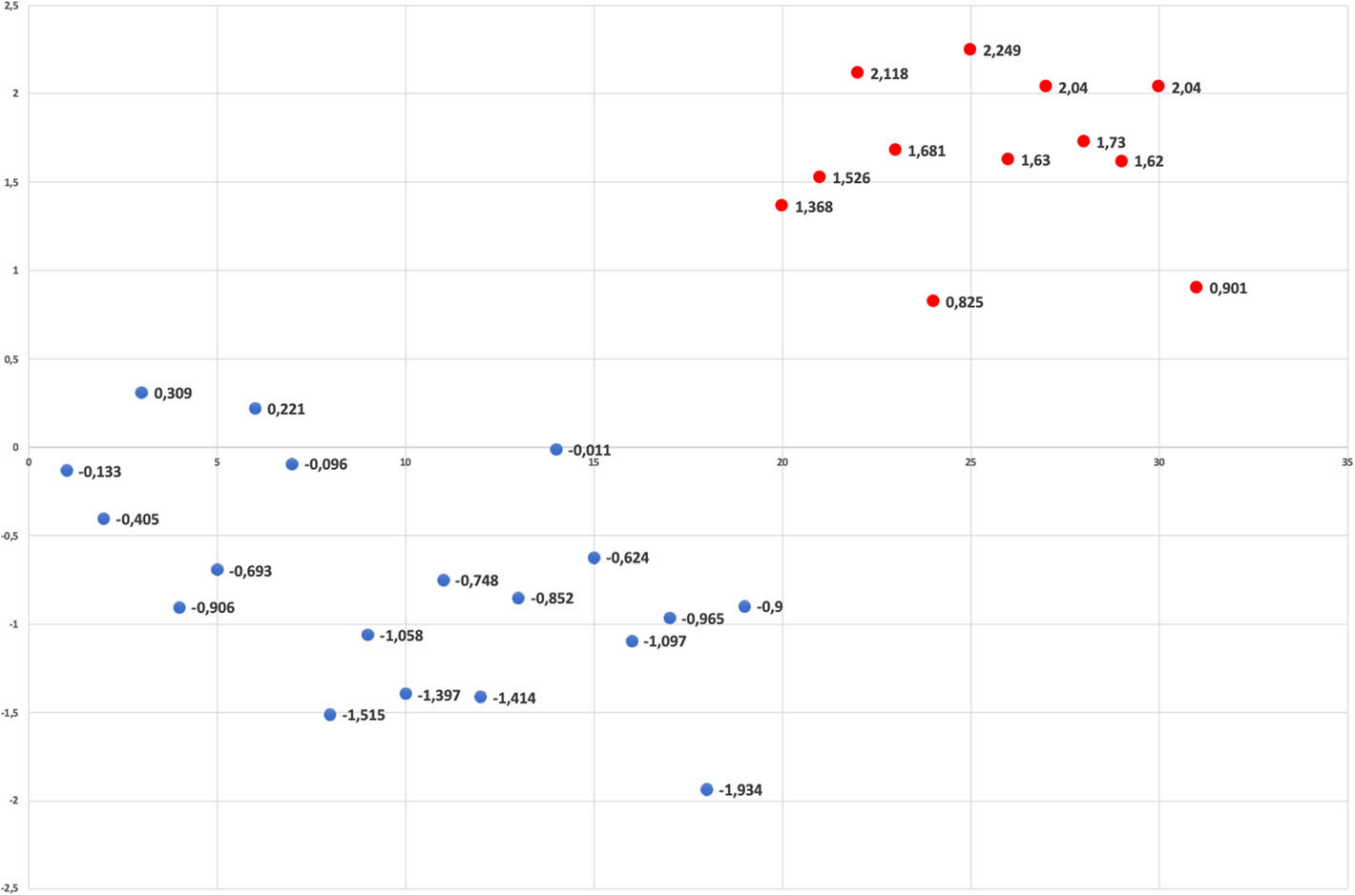
Graphic representation of discriminant analysis scores: blue points RG; red points SG

These variables maximized the Mahalanobis distance between the two groups, with a Wilks’ lambda of 0.616 (statistical significance = 0.004). As shown in Table 8, RG showed a value of 0.0619 whereas SG showed a value of –0.960. According to the score, 5 cases were redistributed into the more appropriate group. The analysis had a classification accuracy of 80.6% (Fig. 4).

## Discussion

The aim of our study was to assess the morphologic dental arches differences in patients with class III malocclusion by comparing a success and a failure treatment group after early orthopedic therapy (RME/FM/BB). Treatment of class III malocclusion is a challenge in orthodontics, as the result of failure is relatively frequent [32]. Therefore, goals of its early interception are to create favorable conditions for a normal growth, improve occlusal relationships, and facial esthetics [3]. As observed by Lee [33], arches’ size and shape have considerable implications in orthodontic diagnosis and treatment planning, as they affect available space, dental relationship, and stability. In literature, however, few works investigated predictive variables of class III early orthopedic treatment failure on dental arches morphology [21, 22].

In this retrospective study, a sample of 31 patients with class III malocclusion was collected. According to the treatment stability of their early orthopedic therapy (RME/FM/BB) [27], they were divided in two groups, relapse group (RG) and success group (SG). Intragroup and intergroups statistics analysis were made to find morphological differences. The cephalometric analysis on lateral radiographs revealed that at T0 the two groups were comparable with no statistically significant differences. On the contrary, at T1 RG showed a hyperdivergent pattern, while SG a normodivergent one. This outcome agrees with other studies [10–24], which reported that the vertical growth pattern was a predictive variable of treatment stability in class III malocclusion [10–24].

Evaluating the dental arches morphology, the T1-T0 comparison showed both in RG and SG statistically significant increases in the maxillary width measurements. This result is due to the RME treatment, which separated the midpalate suture in a “V”-shaped pattern [34, 35] and determined a significant expansion of the maxillary arch and a decrease in the depth of the palate, as stated by Lione et al. [36]. We also noticed an increase in the measurement of palatal area, although not statistically significant, due to the orthopedic expansion.

Focusing on the mandibular arch, RG showed that lower intercanine and intermolar width increased significantly. In SG, there were no mandibular transversal or sagittal changes. These results agree with the discriminant analysis of Franchi et al. [21], who assessed that the deciduous intermolar width was greater in the unsuccessful group, likewise with Wendl et al. [22], who found greater values in the mandibular transversal width in the failure group.

At T0, RG showed a greater upper intercanine width when compared to SG, while no significant differences were found in the upper intermolar width. This result disagrees with Wendl’s findings [22], which assessed that reduced maxillary intermolar width was an additional potential poor prognosis factor in class III malocclusion treatment. However, the authors did not evaluate the upper anterior transversal width and used different sample inclusion criteria and different protocol therapy (such as chincup). The increased UATW at T0 is a morphologic characteristic of the RG patients resulted from our analysis. To our knowledge, no one evaluated the UATW as a predictive variable of relapse and success in class III malocclusion. The increased UATW in the RG subjects could be a predisposing factor for long-term relapse and failure. Regarding the maxillary sagittal length, at T0, we noted shorter values in RG than SG, while at T1 there was a significant increase only in SG.

To evaluate which morphologic characteristic of the maxillary and mandibular arches could be used as stability predictive factors of early orthopedic treatment of class III malocclusion, a discriminant analysis was made. Other studies performed discriminant analysis [11, 14, 17, 21, 23, 24, 31], but only Franchi et al. [21] described a predictive model using one dental width measurement on deciduous dentition.

Our predictive model aimed to identify good or bad responders to interceptive class III malocclusion treatment (RME/FM/BB) by using dental arches morphology analysis in mixed dentition. Discriminant analysis found two predictive variables of relapse: upper posterior sagittal length and upper anterior sagittal length.

Our results show that before treatment, RG subjects have a shorter and wider maxilla. At the end of the treatment, RG presents no significant improvement on the sagittal length if compared to SG, and treatment relapse in RG is further complicated by the transversal increase in mandibular arch.

Our analysis does not imply that our model is able to classify surgical and non-surgical cases, yet it could help to identify eventually bad responders to therapy. A shorter and wider maxilla could be a predisposing factor for long-term relapse and failure.

The main limitations of this study are its retrospective nature and the small sample size due to the low prevalence of class III malocclusion in Caucasian ancestry (around 4.3% as reported by Perillo et al. [37]) and to the difficulty to follow up patients after the first phase of treatment. Another limitation is the absence of an untreated control group, this for the ethical necessity to treat these patients as soon as possible in order to achieve the orthopedic correction of class III malocclusion [2, 3].

## Conclusions

1. Dental arches analysis is a useful diagnostic tool of stability prediction in the early interceptive class III malocclusion treatment and represents a method to predict relapse.
2. Anterior and posterior maxillary sagittal lengths are two predictors of relapse in early orthopedic class III malocclusion treatment.
3. A shorter and wider maxilla could be a predisposing factor for relapse and failure in early orthopedic treatment of class III malocclusion patients.
4. The relapse patients show a statistically significant increase in the transverse dimension of the mandible. The absence of changes in the mandibular arch could be a predictive factor of treatment success.

## Data Availability

The datasets used and/or analyzed during the current study are available from the corresponding author on reasonable request.

## Abbreviations

CS: Cervical stage
CSV: Cervical stage valuation
RG: Relapse group
SG: Success group
RME: Rapid maxillary expander
FM: Facial mask
BB: Bite block
T0: Pre-treatment phase
T1: Post-treatment phase
